# Access to health literacy best practices and the role of health libraries (BiblioSUS Network BVS) in Brazil

**DOI:** 10.1093/eurpub/ckac129.512

**Published:** 2022-10-25

**Authors:** L Saboga-Nunes, LA Santini, F da Silveira, E Moro, L Estabel

**Affiliations:** 1 Institute of Sociology, University of Education, Freiburg, Germany; 2 Ufrgs, Federal University of Rio Grande do Sul, Rio Grande do Sul, Brazil; 3 Polytechnic Institute of Coimbra, Labinsaúde, Coimbra, Portugal

## Abstract

**Introduction:**

Health Literacy (HL) has received recognition of its role and is proposed as a key element of incrementing wellbeing in public health. In the south, this discussion is engaged but a dialogue north-south needs to be incremented to allow further comprehension and implementation of HL best practices. The BiblioSUS Network in Brazil engaged in this discussion while it aims to expand and democratize access to health promotion & HL best practices, disseminated through the virtual health libraries (BVS). As a distribution model of content production by the Ministry of Health in Brazil it reaches a large audience in the country.

**Methods:**

The target of this research includes BiblioSUS workers, representing the most diverse areas of knowledge and the community served by the Network. Data collection included an online survey on HL using the HLS-EU-BR instrument.

**Results:**

The study involved 717 members of the Network and community users of cooperating libraries. Inadequate (12%) and problematic (36%) HL levels revealed that 48% had low levels of HL. The analysis of the 12 variables to assess community's health information needs, showed that 83% of participants do not use the network for health promotion needs, 70.4% do not use for quality of life purposes, but that 41.4% use it for related diseases issues.

**Discussion:**

This research is the first of its kind and emphasize the need to promote health promotion and HL by several means (e.g. like BiblioSUS Network libraries). Communities need to be reached out to help them make decisions favorable to health in everyday life and settings (e.g. home, community, work). While health promotion and disease prevention are targeted, the Brazilian population need to have easier access to reliable health promotion information, in order to contribute to citizens’ empowerment in health.

